# New Frontiers in Organ Preservation and Hepatoprotection

**DOI:** 10.3390/ijms23084379

**Published:** 2022-04-15

**Authors:** Zoltan Czigany, René Hany Tolba

**Affiliations:** 1 Department of Surgery and Transplantation, University Hospital RWTH Aachen, 52074 Aachen, Germany; zczigany@ukaachen.de; 2 Institute for Laboratory Animal Science and Experimental Surgery, Medical Faculty RWTH Aachen University, 52074 Aachen, Germany

This editorial aims to summarize the 13 scientific articles published in the Special Issue entitled “New Frontiers in Organ Preservation and Hepatoprotection”. A collection of six quality original articles and seven review papers have been published in this successful Special Issue with international collaborators from various countries (Figure 1) [1,2,3,4,5,6,7,8,9,10,11,12,13].

Ischemia-reperfusion injury (IRI) represents a significant risk-factor for inferior outcomes in solid organ transplantation [14]. IRI has been shown to be associated with severe complications such as post-reperfusion syndrome, allograft dysfunction or even allograft rejection [10,14]. Over the last few decades, several methods have been developed aiming for hepatoprotection in different clinical and experimental settings [4,10,14,15,16,17,18,19,20,21]. Although accumulating evidence shows the potential positive effects of various protective strategies, multiple challenges remain to be solved.

Out of six original articles, three address the topic of static cold storage [4,5,13]. The experimental study by Di Pasqua et al. has shown the effects of the blockade of mGluR5 by 2-methyl-6(phenylethynyl)pyridine (MPEP) in hepatic preservation injury in rat livers following donation after circulatory death (DCD) [5]. The authors have given MPEP or vehicle 30 min before portal clamping and added this to the cold storage solution. The main finding was a reduced apoptosis after MPEP treatment. One limitation of this study is that no in vivo transplantation of these organs has been carried out [5].

In a further study by our own group, we have investigated the effects of the stimulation of the adenosine A2a receptor in a porcine model of orthotopic liver transplantation and DCD [4]. Animals in the treatment group received the selective adenosine receptor agonist CGS 21680, which was added to the cold flush during retrieval. This study could show that the ex vivo administration of adenosine A2a receptor agonist during the back-table flush mitigates IRI-mediated tissue damage and improves functional graft recovery and survival in a large animal model of DCD liver transplantation.

A study by the Graz group was carried to improve rat uterus preservation. The authors evaluated the effects of Custodiol-N in uterus prolonged cold preservation time (8 and 24 h), compared to Custodiol® solution [13]. The main finding of the study was the superiority of the Custodiol-N solution for uterus graft preservation when compared to standard Custodiol®, which was most likely achieved via the inhibition of oxidative stress and tissue edema [13].

Sulforaphane (SFN) is a naturally occurring isothiocyanate which has been shown to exhibit anti-inflammatory characteristics and reduce platelet activation and inhibit leukocyte adhesion [2]. The Muenster group has designed a murine model to investigate the protective effects and mechanism of action of SFN in intestinal IRI [2]. Acute mesenteric occlusion was modelled by superior mesenteric artery occlusion for 30 min, followed by reperfusion for 2 h, 8 h or 24 h in male wild-type C57BL/6J mice. Interestingly, SFN showed protection with less damage of the intestinal structures observed in histopathological and ultrastructural evaluation. The authors concluded that SFN may be used as a potential therapeutic strategy against intestinal IRI [2].

The only clinical study of this Special Issue was published by the group of Prof. Scherer from Regensburg [6]. They analyzed standard liver biopsies from 46 patients taken at the end of back table preparation and 2 hours after reperfusion following liver transplantation and showed that early IRI was present after 2 hours in 63% of cases [6]. They could observe inferior outcomes in marginal organs which prompted a further comparison between their pre- and post-reperfusion biopsies. This has revealed that transplants with IRI demonstrated significantly more T cell infiltration. Molecular analyses found higher mRNA expression levels of CXCL-1, CD3 and TCRγ locus genes in grafts with significant IRI. Based on these data, they concluded that steatosis exacerbates early IRI by enhancing effector immune cell infiltration [6].

The last original paper in this Special Issue was published by the group of Prof. Yuzo Yamamoto and dealt with the underlying mechanism of rapid liver hypertrophy triggered by associating liver partition and portal vein ligation for staged hepatectomy (ALPPS) in a rat model [11,22]. Briefly, this elegant experimental study has shown that the increase in inflammatory cytokines, such as IL-6 after ALPPS alone, was not enough to produce accelerated hypertrophy [11]. They have postulated that the JAK2/STAT3 pathway might play a crucial role in the additional increase in the liver volume in ALPPS over portal vein ligation (PVL) but not in the basal hypertrophy produced by PVL alone [11]. Furthermore, the expression of Reg3 and Reg3 in the remnant liver was specific to ALPPS animals; thus, this could play a significant role in rapid liver in conjunction with an activation of the JAK2/STAT3 pathway [11].

The seven review pieces published in this Special Issue cover a wide range of topics which are currently under the spotlight of scientific interest within the community [1,3,7,8,9,10,12].

The review paper by Horvath et al. discusses the mitochondrial effects of organ preservation techniques in liver transplantation combining the elements of a narrative review with a systematic review and network meta-analysis [7]. With the ever-increasing significance of dynamic organ preservation and machine perfusion, [16] this review provides a great overview on these topics.

Operational tolerance is one of the ultimate goals in solid organ preservation and immunosuppression and a major focus of research for several decades [23]. Cvetkovski et al. have reviewed protocols for active tolerance induction in liver transplantation, with a focus on identifying tolerogenic cell populations, as well as barriers to tolerance [3]. Furthermore, they have proposed the use of novel immunosuppressive agents to promote immunomodulatory mechanisms favoring tolerance [3].

In a collaborative review paper by our group and the group of Prof. Nemeth, we have comprehensively discussed and reviewed hepatic IRI from the aspect of hemo-rheology and microcirculation [10].

The Kyoto group of Yagi et al. has published a comprehensive and up-to-date review on liver regeneration in the setting of partial liver transplantation and liver resection [12]. They summarized not just the molecular mechanisms but also described the clinical conditions that negatively, or sometimes positively, interfere with liver regeneration [12].

Multi-omics approaches and metabolic profiling are dynamically evolving in translational research in solid organ transplantation [23]. Kvietkauskas et al. from Austria have reviewed the currently available literature on the use of metabolomics in solid organ transplantation, with a special focus on metabolic profiling during graft preservation to assess organ quality prior transplant [8].

The modified Histidine–Tryptophan–Ketoglutarate (HTK) solution, named HTK-N or Custodiol-N, holds promise to improve cold storage outcomes in various settings [9]. The composition of HTK-N differs from the standard HTK solution, carrying larger antioxidative capacity, among other features [9]. Mohr et al. have reviewed the in vitro and in vivo effects of HTK-N [9].

Finally, the last basic science review from Australia is addressing necroptosis, a regulated form of cell death, in the context of liver IRI and organ transplantation focusing predominantly on steatotic livers [1].

Overall, this Special Issue has covered various topics of pharmacological conditioning, aspects of organ preservation and IRI in organ transplantation. Even though this Special Issue was focusing on liver transplantation and hepatic IRI, a broader spectrum of organ transplantation was covered (e.g., articles on uterus transplantation, intestinal ischemia, preservation of solid organs in general). New mechanistic findings on liver regeneration and IRI were reported and summarized in review articles. We hope this Special Issue attracts the attention and interest of the scientific community and inspires researchers to continue the exploration of the scientific topics addressed here.

## Figures and Tables

**Figure 1:**
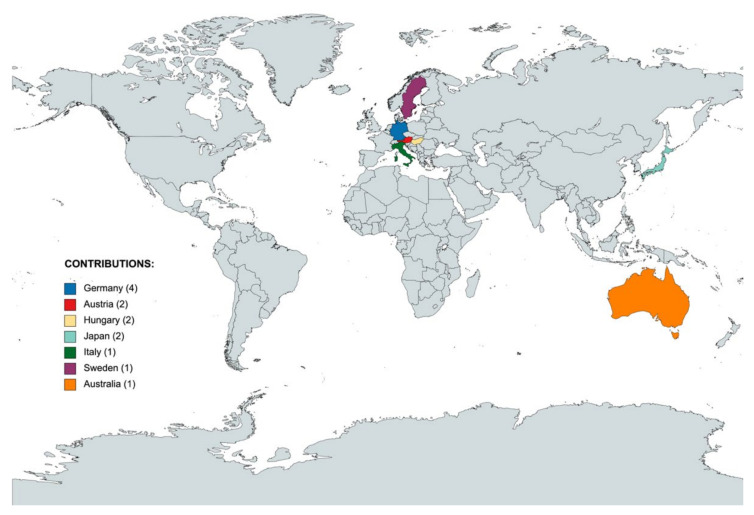
Affiliation of the corresponding authors who published a research or review article in the Special Issue “New Frontiers in Organ Preservation and Hepatoprotection”. This map shows an international representation with the contribution from multiple European countries, as well as from Japan and Australia.
